# Meta-analysis: preoperative transcatheter arterial chemoembolization does not improve prognosis of patients with resectable hepatocellular carcinoma

**DOI:** 10.1186/1471-230X-13-51

**Published:** 2013-03-19

**Authors:** Yanming Zhou, Xiaofeng Zhang, Lupeng Wu, Feng Ye, Xu Su, Lehua Shi, Bin Li

**Affiliations:** 1 Department of Hepatobiliary & Pancreatovascular Surgery, First affiliated Hospital of Xiamen University; Oncologic Center of Xiamen, Xiamen, China; 2 Department IV of Hepatic Surgery, Eastern Hepatobiliary Surgery Hospital, Second Military Medical University, Shanghai, 200438, China

**Keywords:** Hepatocellular carcinoma, Transcatheter arterial chemoembolization, Prognosis

## Abstract

**Background:**

Long-term outcomes of partial liver resection of hepatocellular carcinoma (HCC) remain satisfactory due to high incidences of recurrence. This study was intended to see whether preoperative transcatheter arterial chemoembolization (TACE) reduces postoperative tumor recurrences and prolongs survival of patients with resectable HCC.

**Methods:**

A computerized literature search was performed to identify relevant articles. The quality of nonrandomized comparative studies (NRCTs) was assessed using the methodological index for nonrandomized studies (MINORS). Data synthesis was performed using Review Manager 5.0 software.

**Results:**

Twenty-one studies (4 randomized controlled trials and 17 NRCTs) with a total of 3,210 participants were suitable for analysis. There was no significant difference in disease-free and overall survival at 5-year (32.1% vs. 30.0% and 40.2% vs. 45.2%), and intra- and extra-hepatic recurrence (51.2% vs.53.6% and 12.9% vs.10.3%) between patients with and without preoperative TACE. Postoperative morbidity (28.9% vs. 26.8%) and in-hospital mortality (4.1% vs. 3.1%) were also similar between the two groups.

**Conclusions:**

Preoperative TACE does not seem to improve prognosis and therefore it is prudent to recommend it as a preoperative routine procedure for resectable HCC.

## Background

Hepatocellular carcinoma (HCC) is the fifth most common malignancy worldwide, and ranks the third leading cause of cancer-related death [1]. Hepatectomy is considered the main curative treatment for HC with a 5-year survival of 23.8–54.8% as reported in the most recent series [2-5]. Tumor recurrence even after radical surgery affects 75-100% HC patients, accounting for the major cause of death of HC patients [6]. The development of appropriate strategies to prevent tumor recurrence is therefore critical for improving long-term outcomes of HC patients after surgical resection.

Transcatheter arterial chemoembolization (TACE), which induces tumor ischemic necrosis by arterial injection of chemotherapeutic drugs and embolizing agents, is recommended as the first-line palliative treatment for inoperable HCC in the 2005 practice guidelines issued by the American Association for the Study of Liver Diseases [7]. Some researchers believed that it may reduce the viability of HCC cells before surgery and reduce postoperative tumor recurrence [8]. Although several studies have demonstrated the survival benefits of preoperative TACE for patients with HCC [9,10], others have failed to show any significant survival benefit [11-16]. Therefore, the role of preoperative TACE for HCC remains a contradictory issue. A recent review of three randomized controlled trials (RCTs) showed that preoperative TACE did not significantly improve survival [17]. However, their study only involved 257 participants, and therefore may not be convincing enough to confirm the effect conclusively [18]. To produce more reliable evidence for clinical decision-making, meta-analytic techniques could be applied to nonrandomized comparative studies (NRCTs) to ensure both the number and sample size of RCTs [19], knowing that meta-analysis of well-designed NRCTs is feasible and the results are remarkably similar to those of contemporaneous RCTs [20]. The present meta-analysis should be able to provide an updated evaluation on preoperative TACE for resectable HCC by taking into account all the currently evidence from RCTs and well-designed NRCTs.

## Methods

### Inclusion and exclusion criteria

The present meta-analysis was performed by following the recommendations of the Preferred Reporting Items for Systematic Reviews and Meta-Analyses (PRISMA) Statement. Primary studies that evaluated the efficacy of preoperative TACE vs. no-preoperative TACE for resectable HCC were considered for inclusion. For duplicate publications reported by the same authors, either the one of higher quality or the most recent publication was selected. Abstracts, letters, editorials and expert opinions, reviews without original data, case reports, and uncontrolled studies were excluded. Study populations including recurrent HCC or unresectable diseases were also excluded.

### Study selection

A computerized search of the literature was performed by searching Medline, EMBASE, OVID, and Cochrane database from the time of inception to June 2012. The following medical subject heading (MeSH) terms were used: “hepatectomy,” “hepatocellular carcinoma,” and “transarterial chemoembolization”. Only studies on humans and in the English language were considered for inclusion. Reference lists of all identified articles were manually searched for additional studies. Publication bias was assessed visually using a funnel plot.

### Data extraction

Two reviewers (LW and YZ) independently extracted the following parameters from each study: first author, year of publication, study population characteristics, study design, inclusion and exclusion criteria, the number of subjects in each arm, survival, recurrence, morbidity and mortality. All relevant texts, tables and figures were reviewed for data extraction.

### Qualitative analysis

The RCTs were scored using the Jadad composite scale [21], which evaluates studies based on appropriate randomization, double blinding, and an adequate description of withdrawals and drop-outs. For evaluation of NRCTs, the methodological index for nonrandomized studies (MINORS) with some modifications were applied [22]. The following 12 items were evaluated for each study: a clearly stated aim, consecutive patients, prospective data collection, reported endpoints, unbiased outcome evaluation, adequate length of follow-up, loss to follow up <5%, ≥ 20 patients in each arm, adequate control group, contemporary groups, controls equivalent to cases, and adequate statistical analyses. Studies achieving more than 16 points (maximum 24) were defined as well-designed and were included in the meta-analysis [20]. Those with less than 16 points were excluded.

### Outcomes of interests

Items for assessing long-term outcomes included disease-free and overall survival rate at 5 year, total recurrence, hepatic recurrence, and extrahepatic recurrence.

Items for assessing short-term outcomes included postoperative morbidity and in-hospital mortality.

### Statistical analysis and synthesis

Dichotomous variables were tested by odds ratio (OR) with a 95% confidence interval (95% CI), and continuous variables were tested by the weighted mean difference (WMD) with a 95% CI. Between-study heterogeneity was evaluated by *χ*^2^ and *I*^*2*^. Data that were not significantly heterogeneous *(P* > 0.1) were calculated using a fixed effects model, and heterogeneous data (*P* < 0.1) were calculated using a random-effects model. Sensitivity analysis was undertaken by using the following subgroups: (1) RCTs, (2) NRCTs, and (3) studies with matched clinicopathological parameters. Statistical analyses were performed with Review Manager version 5.0 (The Cochrane Collaboration, Software Update, Oxford). A value of *P* < 0.05 was considered statistically significant.

## Results

### Eligible studies

Twenty-six studies that matched the selection criteria were retrieved from the electronic databases [9-16,23-41]. Of these studies, one study was excluded due to overlap of authors and nonrandomized nature [23], and therefore the most recent RCT was included [24]. Four NRCTs with fewer than 16 points were excluded [9,25-27]. Finally, 21 articles, including four RCTs [14-16,24] and 17 NRCTs [10-13,28-40], met the inclusion criteria.

The characteristics of the 21 studies are summarized in Table 1. A total of 3,210 patients were included in the meta-analysis, of whom 1,431 received preoperative TACE and 1,779 were allocated to a control group. The number of patients in each study varied from 40 to 350 patients. In the enrolled patients, the percentage of men ranged from 76.9% to 100% and the mean age ranged from 45.3 ± 9.8 to 68.1 ± 5.7 years. Eight studies were completely matched with respect to the reported clinicopathological parameters [12,15,16,28,30,32,35,39]. One trial was conducted to compare outcomes of whole-liver TACE, selective TACE, and without preoperative TACE [24]. Only selective TACE arm was considered in the present meta-analysis.

**Table 1:** Clinical background of studies included in the meta-analysis

**Reference**	**Year**	**Inclusion Period**	**Country**	**Group**	**No. of patients**	**M/F**	**Mean age (years)**	**Mean AFP (ng/ml)**	**Child-Pugh A/B/C**	**LB N/H/C**	**Tumor size (cm)**	**Matching**	**Not matching**	**Score**
**RCTs**														
Wu [14]	1995	1983-1991	Taiwan	TACE (+) TACE (−)	24 28	23/1 23/5	51.8 ± 12.4 53.2 ± 11.5	≥ 400 (n = 15) ≥ 400 (n = 16)	22/2/0 24/4/0	—/—/14 —/—/12	14.3 ± 4.2 14.5 ± 3.3	1-3,5-15	17	2
Yamasaki [15]	1996	1987-1989	Japan	TACE (+) TACE (−)	50 47	50/0 47/0	54.9 ± 6.4 57.1 ± 4.9	— —	— —	—/—/— —/—/—	3.1 ± 0.8 3.3 ± 0.9	1,2,4,9-12	—	2
Zhou [16]	2009	2001-2003	China	TACE (+) TACE (−)	52 56	48/4 49/7	45.3 ± 9.8 46.8 ± 9.6	1244.2 ± 376 1387.5 ± 426	44/8/0 54/2/0	—/—/49 —/—/50	9.0 ± 3.2 9.5 ± 3.9	1-3,5-10, 12,14,15	—	3
Kaibori [24]	2012	2004-2007	Japan	TACE (+) TACE (−)	42 43	35/7 32/11	68.1 ± 5.7 66.1 ± 10.6	2432 ± 11638 858 ± 5269	37/5/0 39/4/0	1/27/14 4/28/11	4.3 ± 2.1 4.8 ± 4.1	1-10,12, 13,15	11	3
**NRCTs**														
Nagasue [12]	1989	1980-1986	Japan	TACE (+) TACE (−)	31 107	25/6 90/17	56.5 ± 9.1 59.4 ± 8.9	>20 (n = 18) >20 (n = 76)	17/13/1 69/32/6	—/—/26 —/—/88	>3 (n = 17) >3 (n = 72)	1-3,5,6, 8–11,14	—	19
Adachi [13]	1993	1981-1991	Japan	TACE (+) TACE (−)	46 26	39/7 21/5	55.9 ± 7.71 59.8 ± 5.7	>20 (n = 24) >20 (n = 15)	— —	—/—/29 —/—/22	2.6 ± 0.9 2.1 ± 0.9	2-4,6-8,11,14,16	1,9,13	18
Harada [28]	1996	1982-1994	Japan	TACE (+) TACE (−)	105 35	90/15 30/5	57.6 ± 9.7 59.8 ± 9.9	— —	— —	—/—/69 —/—/24	>5 (n = 36) >5 (n = 9)	1,2,4,7-10,12	—	19
Uchida [29]	1996	1986-1991	Japan	TACE (+) TACE (−)	60 68	50/10 52/16	59.0 ± 9.9 62.0 ± 7.2	>20 (n = 27) >20 (n = 45)	37/18/5 45/18/5	—/—/42 —/—/49	3.7 ± 3.1 4.4 ± 3.4	2,4-10,14	1,3,11,13	18
Majno [10]	1997	1985-1995	France	TACE (+) TACE (−)	49 27	44/5 26/1	59.2 ± 7.1 60.9 ± 7.8	285 ± 1629 129 ± 250	43/6/0 17/10/0	0/0/49 0/0/27	5.05 ± 2.5 3.9 ± 1.8	1-3,8,10, 12-14	5-7	18
Di Carlo [11]	1998	1989-1997	Italy	TACE (+) TACE (−)	55 45	49/6 34/11	63.0 ± 6.0 62.0 ± 6.0	>10 (n = 13) >100 (n = 6)	48/—/— 36/—/—	0/0/55 0/0/45	>3 (n = 32) >3 (n = 31)	1-3,5,7-12,14,15	13	18
Paye [30]	1998	1986-1992	France	TACE (+) TACE (−)	24 24	21/3 17/7	57 ± 2 54 ± 3	2560 (2–46000) 4229 (2–73000)	22/2 22/2	1/—/13 1/—/13	7.8 ± 1 7.3 ± 1	1-3,5-10,12-14,16	—	17
Lu [31]	1999	1988-1994	China, Japan	TACE (+) TACE (−)	44 76	36/8 57/19	51.5 54.5	— —	31/13/0 61/15/0	—/—/28 —/—/44	7.3 7.6	2,5,6,8,9,11,14	1	20
Ochiai [32]	2003	1978-1994	Japan	TACE (+) TACE (−)	100 48	78/22 43/5	59.6 ± 8 58.9 ± 8.7	>400 (n = 74) >400 (n = 30)	— —	—/—/— —/—/—	4.4 ± 3.3 4.4 ± 3.1	1-4,6,7,9, 10,12-15	—	20
Sugo [33]	2003	1997-2000	Japan	TACE (+) TACE (−)	146 81	122/24 67/14	57.6 ± 9.4 60.5 ± 9	— —	118/27/1 74/6/1	—/—/116 —/—/58	4.5 ± 3.0 4.8 ± 3.9	2,5,8,9	1	18
Sasaki [34]	2006	1982-2003	Japan	TACE (+) TACE (−)	109 126	85/24 97/29	>65 (n = 38) >65 (n = 68)	>100 (n = 40) >100 (n = 49)	— —	—/—/76 —/—/61	≥5 (n = 27) ≥5 (n = 47)	2-4,7,9,11, 12	1,8	20
Chen [35]	2007	1990-2004	China	TACE (+) TACE (−)	89 157	71/18 136/21	45.5 ± 6.3 48.6 ± 5.7	2838 ± 1721 2335 ± 1088	78/11/0 142/15/0	—/—/15 —/—/25	9.5 ± 2.6 9.9 ± 3.1	1-3,6-10,12,13,16	—	20
Choi [36]	2007	1998-2005	Korea	TACE (+) TACE (−)	120 153	93/27 117/36	52.4 ± 9.6 52.4 ± 10.4	8094 ± 68967 2292 ± 7444	117/0/0 150/2/0	—/—/56 —/—/75	>5 (n = 44) >5 (n = 50)	1-6,8-10,12-14	7	19
Kim [37]	2008	1995-2000	Korea	TACE (+) TACE (−)	97 237	80/17 194/43	48.8 ± 9.2 51.7 ± 10.2	>1000 (n = 70) >1000 (n = 31)	97 237	—/—/77 —/—/182	>5 (n = 49) >5 (n = 101)	1-3,5-10,12,14	16	19
Lee [38]	2009	2000-2006	Taiwan	TACE (+) TACE (−)	114 236	89/25 173/63	57.9 ± 11.3 57.8 ± 11.7	2265.88 ± 8438.55 1862.52 ± 1388.63	110/4/0 232/4/0	—/—/79 —/—/152	4.3 ± 3.1 3.9 ± 2.7	1-3,5,7-10, 12,15	11	18
Kang [39]	2010	1997-2007	Korea	TACE (+) TACE (−)	32 64	25/7 51/13	52.4 ± 9.8 54.0 ± 10.8	9584.4 ± 26238.5 5100.9 ± 24773.9	30/2/0 64/0/0	—/—/— —/—/—	4.3 ± 2.5 4.5 ± 3.2	1-3,5,6,9,10,15	—	20
Yamashita [40]	2012	1995-2008	Japan	TACE (+) TACE (−)	42 95	36/7 77/18	58 ± 12 64 ± 9	1527 ± 4415 1125 ± 4713	31/—/— 88/—/—	—/—/7 —/—/26	9.1 ± 3.2 7.9 ± 3.0	2-4,7-11	1,5,6	20

LB liver background; N/H/C normal/hepatitis/cirrhosis; 1, Age; 2, Gender; 3, AFP level; 4, ICG R15 (%); 5, Child-Pugh grade; 6, Viral status; 7, Operative procedures; 8, LB; 9, Tumor size; 10 Tumor number;11, Histologic grade; 12,Vascular invasion; 13, Tumor encapsulation; 14, Intrahepatic metastasis or satellite nodules; 15, Pathological stage; 16,Capsular invasion;17, Adjacent organ invasion.

The data regarding the effects of TACE on tumor responses were available in 18 studies [10-16,24,28,30-38], in which histological examination on surgical specimens revealed that a total of 260 patients (20.1%) of 1,292 patients had complete tumor necrosis ranging from 0 to 53.6%.

**Figure 1:**
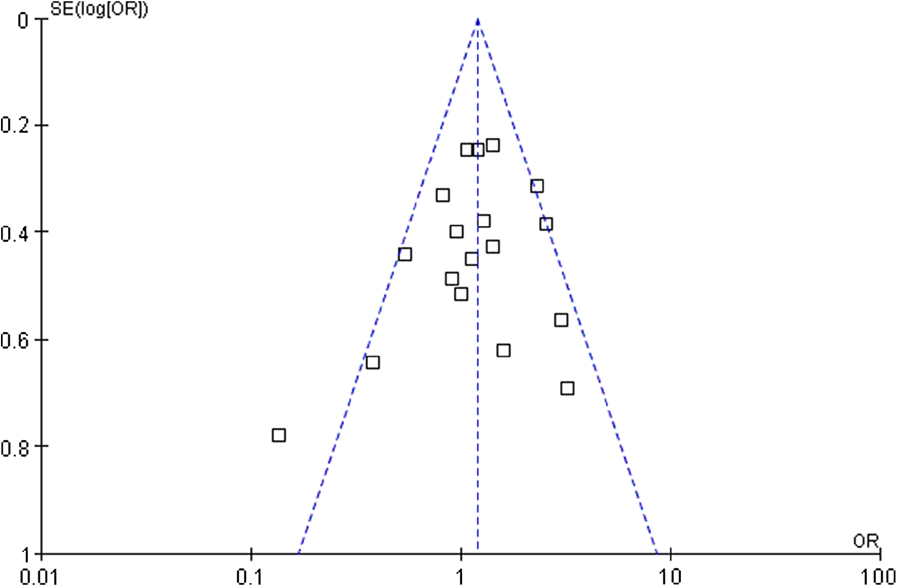
**Funnel plot analysis of publication bias.** The outcome was the 5-year disease-free survival.

The funnel plot (Figure 1) for 5-year disease-free survival in the included studies demonstrated asymmetry, indicating an insignificant sign of publication bias.

### Overall meta-analysis

The results from overall meta-analysis are summarized in Table 2.

**Table 2:** Results of overall meta-analysis

**Outcomes**	**No. of studies**	**No.of patients**		**Results**		**OR (95% CI)**	***P *value**	**HG *p *value**
		**TACE (+)**	**TACE (−)**	**TACE (+)**	**TACE (−)**			
**Long-term outcomes**								
5-years disease-free survival	18[10,11,13-16,24,28,31-40]	1275	1567	32.1%	30.0%	1.19 (0.93, 1.53)	0.17	0.02
5-years overall survival	16[11,12,14-16,24,28,29,31,34-40]	1038	1463	40.2%	45.2%	0.85 (0.59, 1.22)	0.37	<0.01
Total recurrence	11[10,14,16,24,30,32,35,36],[38-40]	673	917	61.0%	58.4%	0.99 (0.72, 1.36)	0.95	0.08
Intrahepatic recurrence	12[10-12,14,16,24,29,30,32,35],[36,39]	660	797	51.2%	53.6%	0.84 (0.67, 1.05)	0.12	0.23
Extrahepatic recurrence	9[10,14,16,24,30,32,35,36],[39]	519	591	12.9%	10.3%	1.30 (0.88, 1.92)	0.19	0.33
**Short-term outcomes**								
Overall morbidity	11[10-12,14,16,24,29,30,35,36],[40]	583	803	28.9%	26.8%	1.02 (0.80, 1.32)	0.85	0.42
Liver failure	6[11,12,14,16,29,36]	337	457	5.9%	6.3%	1.06 (0.57, 1.96)	0.86	0.31
Bile leakage	6[12,14,16,29,35,36]	371	569	3.5%	2.8%	1.12 (0.53, 2.35)	0.77	0.80
Pleural effusion	4[12,29,35,36]	300	485	7.0%	8.0%	0.93 (0.53, 1.65)	0.24	0.26
Ascites	3[29,35,36]	269	378	6.3%	6.1%	0.98 (0.51, 1.89)	0.96	0.16
Intra-abdominal abscess	5[11,14,29,35,36]	348	451	2.5%	1.3%	1.66 (0.63, 4.40)	0.31	0.58
Wound infection	5[12,14,16,35,36]	311	501	3.2%	2.5%	1.11 (0.48, 2.53)	0.81	0.35
Postoperative bleeding	3[11,12,36]	206	305	3.3%	2.9%	1.25 (0.41, 3.81)	0.69	0.63
Stress ulcer bleeding	3[12,29,35]	180	332	1.1%	1.2%	1.31 (0.29, 5.92)	0.73	0.74
Pneumonia	4[11,12,14,35]	199	337	4.0%	2.1%	1.64 (0.60, 4.46)	0.33	0.23
Mortality	16[10-12,14-16,24,28-30,33-36,38,40]	1100	1325	4.1%	3.1%	1.25 (0.80, 1.97)	0.33	0.85

HG heterogeneity.

### Long-term outcomes

**Figure 2:**
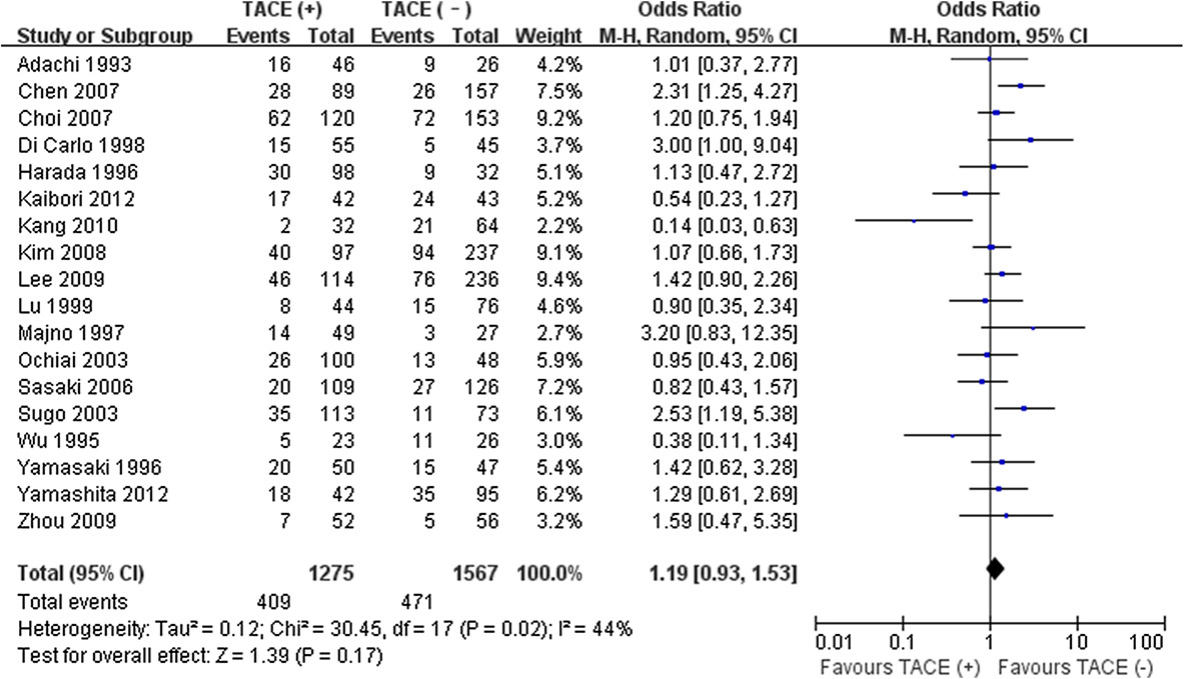
Results of the meta-analysis on 5-year disease-free survival.

**Figure 3:**
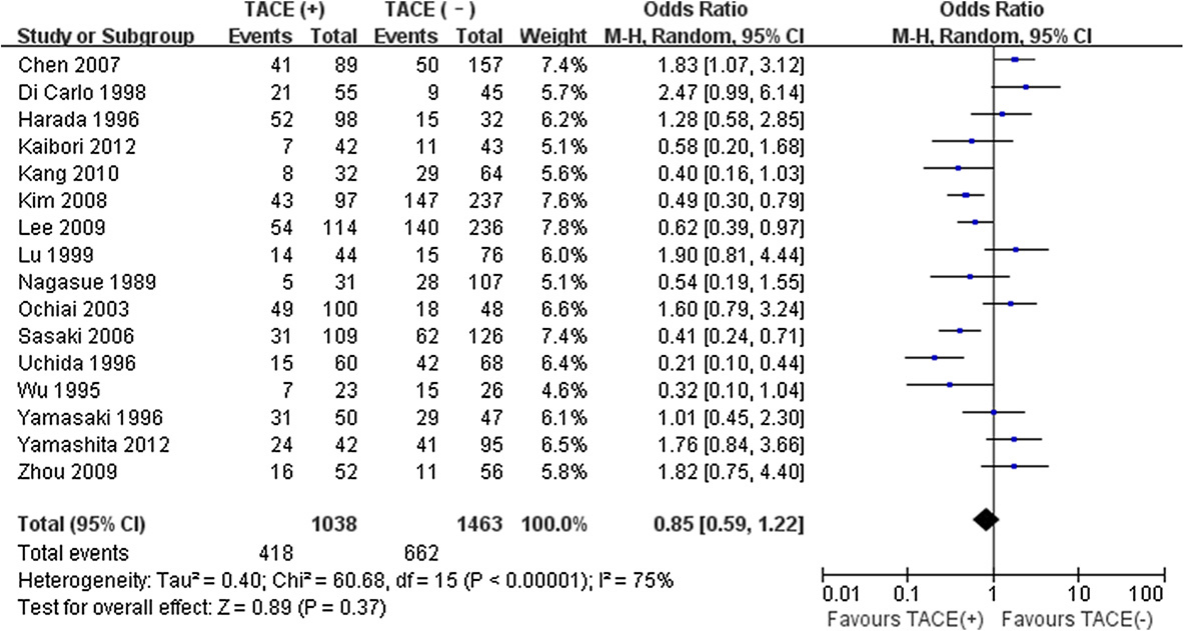
Results of the meta-analysis on 5-year overall survival.

The 5-year disease-free survival was 7.0–57% for preoperative TACE and 8.0–48.8% for control in 18 studies [10,11,13-16,24,28,31-40]. The 5-year overall survival was 15.4–62.7% for preoperative TACE and 19.0–62.5% for control in 16 studies [11,12,14-16,24,28,29,31,34-40]. Pooled analyses showed that preoperative TACE use was not associated with significant improvement in disease-free and 5-year overall survival (32.1% vs. 30.0%, *P* = 0.17; 40.2% vs. 45.2%, *P* = 0.37, respectively). There was significant heterogeneity between studies reporting these two outcomes (*P* = 0.02, *P* < 0.01; respectively) (Figures 2, 3).

Eleven studies reported on total recurrence after surgery: 411 61.0%) of 673 with preoperative TACE vs.536 (58.4%) of 917 without TACE [10,14,16,24,30,32,35,36],[38-40]. Pooled analyses showed that the difference was insignificant between the two groups (*P* = 0.95). There was moderate heterogeneity between studies (*P* = 0.08).

**Figure 4:**
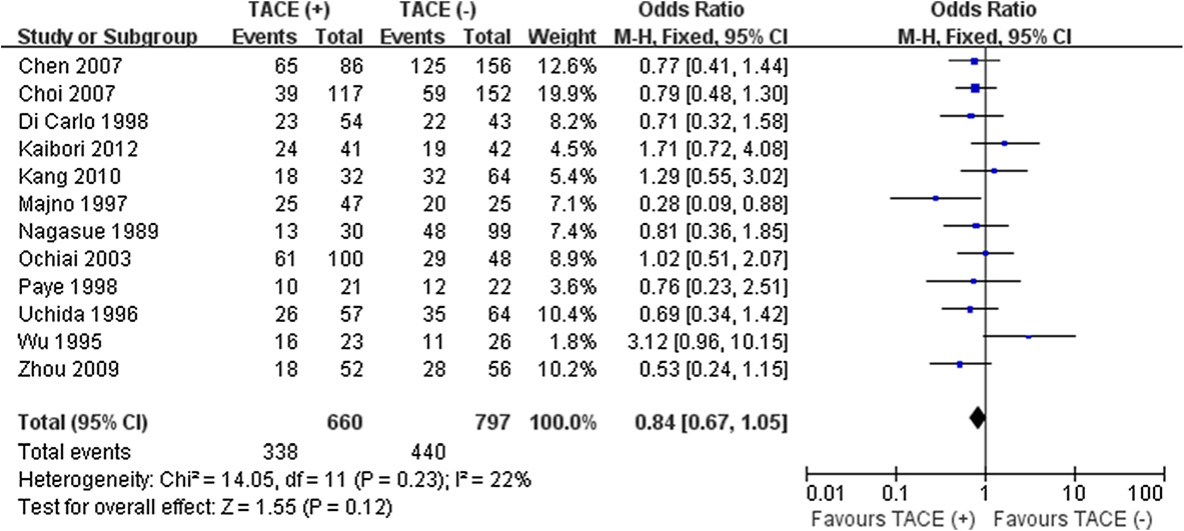
Results of the meta-analysis on intrahepatic recurrence.

**Figure 5:**
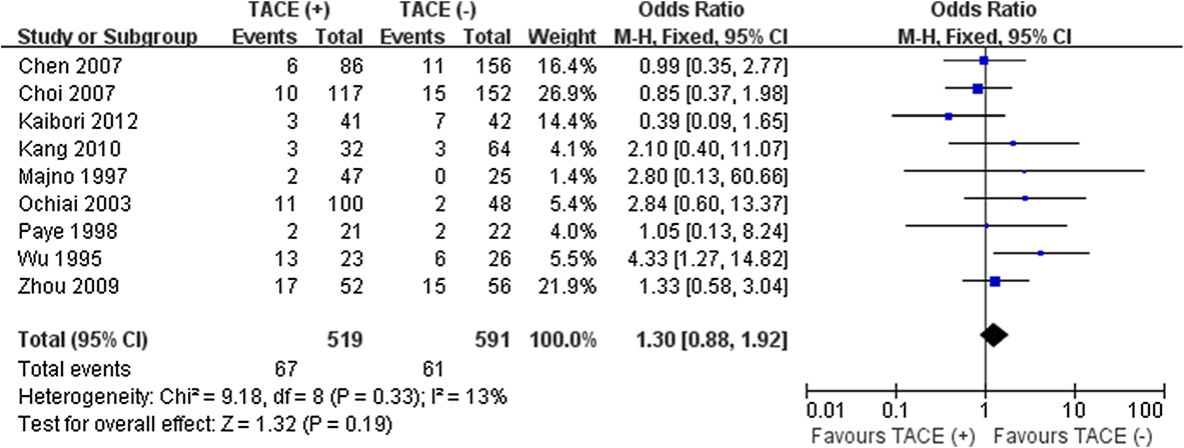
Results of the meta-analysis on extrahepatic recurrence.

Further pooled analysis of studies providing information found that the percentages of both intra- and extrahepatic recurrence were also similar in the two groups (51.2% vs.53.6%, *P* = 0.12; 12.9% vs. 10.3%, *P* = 0.19, respectively). No significant heterogeneity was detected between the groups in reporting these two outcomes (Figures 4, 5).

### Short-term outcomes

**Figure 6:**
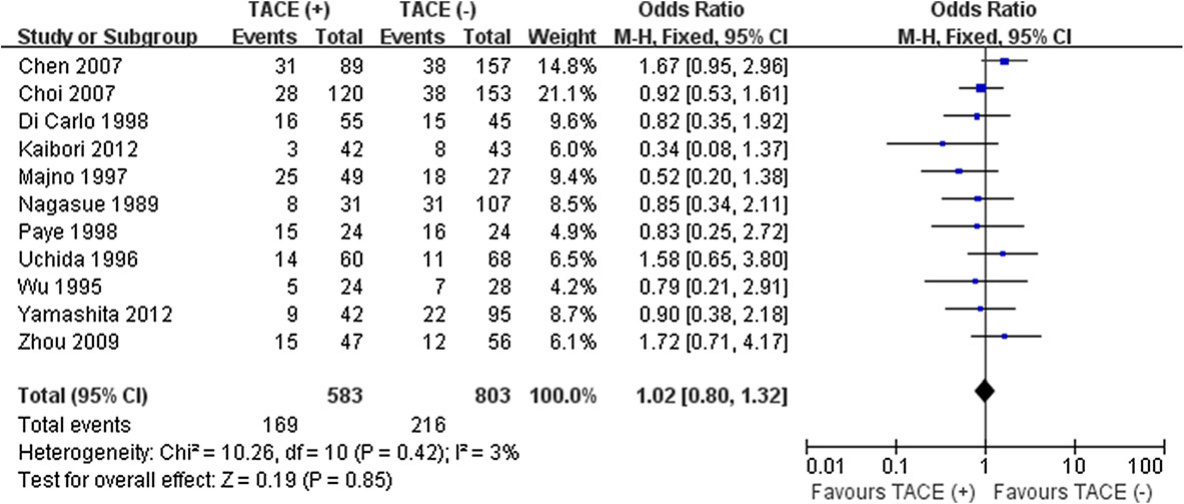
Results of the meta-analysis on overall morbidity.

Eleven studies reported on overall morbidity: 169 (28.9%) of 583 with preoperative TACE vs. 216 (26.8%) of 803 without TACE [10-12,14,16,24,29,30,35,36],[40]. Pooled analyses showed that the difference was insignificant between the two groups (*P* =0.85) without significant heterogeneity (Figure 6). Further subanalysis showed that the risk was comparable between patients in both study groups with respect to liver failure (5.9% vs. 6.3%, *P* =0.86), bile leakage (3.5% vs. 2.8%, *P* =0.77), pleural effusion (7.0% vs. 8.0%, *P* =0.24), ascites (6.3% vs. 6.1%, *P* =0.96), intra-abdominal abscess (2.5% vs. 1.3%, *P* =0.31), wound infection (3.2% vs. 2.5%, *P* =0.81), postoperative bleeding (3.3% vs. 2.9%, *P* =0.69), stress ulcer bleeding (1.1% vs. 1.2%, *P* =0.73), and pneumonia (4.0% vs. VS. 2.1%, *P* =0.33).

**Figure 7:**
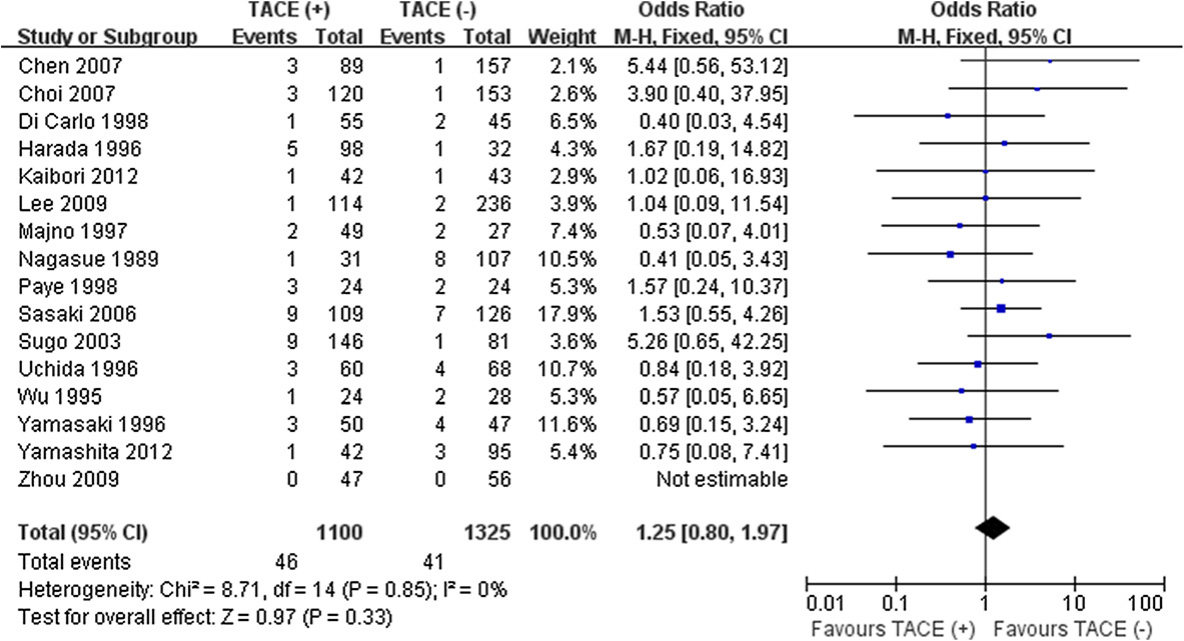
Results of the meta-analysis on in-hospital mortality.

Sixteen studies reported on in-hospital mortality [10-12,14-16,24,28-30,33-36,38,40], showing that 87 patients died: 46 in the preoperative TACE group and 41 in the control group. Pooled analyses showed that there was no statistical difference between the two groups (*P* =0.33) (Figure 7).

### Sensitivity analysis

As Table 3 shows, the results of sensitivity analysis derived from three subgroups were all consistent with those derived from overall meta-analysis.

**Table 3:** Results of sensitivity analysis

**Outcome**	**No. of studies**	**No.of patients**		**Results**		**OR (95% CI)**	***P *value**	**HG *p *value**
		**TACE (+)**	**TACE (−)**	**TACE (+)**	**TACE (−)**			
**RCTs**								
5-years disease-free survival	4[14-16,24]	167	172	29.3%	31.9%	0.85 (0.53, 1.39)	0.52	0.16
5-years overall survival	4[14-16,24]	167	172	36.5%	38.3%	0.89 (0.56, 1.41)	0.61	0.11
Total recurrence	3[14,16,24]	116	124	74.1%	74.2%	1.03 (0.36, 2.90)	0.96	0.07
Intrahepatic recurrence	3[14,16,24]	116	124	50.0%	46.7%	1.32 (0.47, 3.70)	0.59	0.02
Extrahepatic recurrence	3[14,16,24]	116	124	28.4%	22.5%	1.37 (0.42, 4.43)	0.60	0.04
Overall morbidity	3[14,16,24]	113	127	20.3%	21.2%	0.97 (0.52, 1.82)	0.93	0.14
Mortality	4[14-16,24]	163	174	3.1%	4.0%	0.70 (0.22, 2.30)	0.56	0.95
**NRCTs**								
5-years disease-free survival	14[10,11,13,28,31-40]	1108	1395	32.4%	29.8%	1.28 (0.98, 1.67)	0.07	0.04
5-years overall survival	12[11,12,28,29,31,34-40]	871	1291	40.9%	46.1%	0.86 (0.55, 1.32)	0.48	<0.01
Total recurrence	8[10,30,32,35,36,38-40]	559	795	58.1%	55.8%	0.98 (0.77, 1.25)	0.86	0.11
Intrahepatic recurrence	9[10-12,29,30,32,35,36,39]	546	675	51.2%	56.5%	0.79 (0.62, 1.01)	0.06	0.84
Extrahepatic recurrence	6[10,30,32,35,36,39]	403	467	8.4%	7.0%	1.22 (0.73, 2.06)	0.44	0.74
Overall morbidity	8[10-12,29,30,35,36,40]	470	676	31.0%	27.9%	1.03 (0.79, 1.36)	0.81	0.50
Mortality	12[10-12,28-30,33-36,38,40]	937	1151	4.3%	2.9%	1.39 (0.85, 2.28)	0.19	0.74
**Studies with complete matched clinicopathological parameters**								
5-years disease-free survival	6[15,16,28,32,35,39]	421	404	26.8%	22.0%	1.13 (0.62, 2.05)	0.69	0.02
5-years overall survival	7[12,15,16,28,32,35,39]	452	511	44.6%	35.2%	1.15 (0.76, 1.74)	0.50	0.08
Total recurrence	5[16,30,32,35,39]	297	349	73.7%	76.5%	0.91 (0.62, 1.33)	0.64	0.15
Intrahepatic recurrence	6[12,16,30,32,35,39]	328	456	56.4%	60.1%	0.81 (0.59, 1.11)	0.20	0.69
Extrahepatic recurrence	5[16,30,32,35,39]	297	349	13.1%	9.4%	1.40 (0.83, 2.38)	0.21	0.80
Overall morbidity	4[12,16,30,35]	191	344	36.1%	28.2%	1.36 (0.91, 2.01)	0.13	0.48
Mortality	6[12,15,16,28,30,35]	339	423	4.4%	3.7%	1.16 (0.52, 2.57)	0.71	0.50

HG heterogeneity.

## Discussion

The design of TACE is based on the principle that primary HCC is supplied almost exclusively (90%) by the hepatic arteries. The obstruction of the feeding arteries can induce tumor ischemic necrosis. A combination of chemotherapy can drastically increase the local concentration of the chemotherapeutic agent and may improve the benefit of therapy. In 2003, a review of 7 RCTs showed that TACE significantly improved 2-year overall survival compared with nonactive treatment in patients with unresectable HCC [41]. In 2005, this therapy was recommended as standard intervention for unresectable patients with large/multifocal HCC who do not have vascular invasion or extrahepatic spread [7]. In contrast, the results of present pooled analysis of 21 trials do not support the use of preoperative TACE in the management of patients with resectable HCC.

Although TACE is effective for main tumors, intrahepatic metastases, tumor thrombi in the portal veins, and capsular invasion, which are considered risk factors contributing to HCC recurrence, are more unresponsive to TACE because of collateral and portal vein blood supply [8,13,16]. In addition, TACE mainly affects well-differentiated HCC without completely killing poorly differentiated cells [42], which harbour a high grade of malignancy and ready spread within the portal venous system [43]. Furthermore, hematogeneous and lymphatic spread dissemination of cancer cells can precede TACE treatment. It is therefore reasonable to conclude that TACE is unable to reduce the risk of postoperative recurrence, or confer a survival advantage.

Adachi *et al.*[13] and Harada *et al. *[28] reported that the TACE subgroup with complete tumor necrosis had a better survival rate than the group without TACE. This is partly due to residual confounding, because tumors with complete necrosis are strongly associated with favorable tumor-related factors, such as smaller tumor size and tumor encapsulation or less portal involvement. On the other hand, several other reports failed to make the same conclusion [15,28,30,31]. It was found in the present study that approximately 20.1% of HCC tumors responded completely to TACE, suggesting that most of the HCC tumor cells were viable even when treated with TACE. The labeling index of proliferating cell nuclear antigen (a most widely used proliferation-associated marker) was significantly higher in the TACE group, indicating that residual HCC cells following preoperative TACE exhibit more aggressive behavior [44]. In support of this observation, Liou *et al. *[45] indicated that incomplete HCC necrosis after TACE (especially combined with necrotic area >50% main tumor size) was associated with the development of lung metastasis that has a strong adverse impact on patient survival. Adachi *et al. *[13] and Kim *et al.*[37] found that subjects with partial tumor necrosis had the lowest disease-free survival rate among the TACE subgroups and tended to have a lower survival rate than the group without TACE. Zhou *et al. *[16] noted that five patients lost the chance of potentially curative liver resection because of progression of disease with metastases (n = 4) and liver failure (n = 1) during intervals between last TACE hepatic resection. The mechanism underlying accelerated tumor progression by TACE is unclear. Intratumoral necrosis was found to weaken the adhesive potential of the tumor and subsequently facilitate the release of cancer cells from the primary tumor and dislodgment into the bloodstream [46]. Xiao *et al.*[47] reported that mutated p53 could enhance the proliferation of HCC cells and suppress the apoptosis of HCC cells after TACE. In addition, vascular endothelial growth factor (VEGF), the most specific known angiogenic factor that plays a critical role in tumor growth, invasion, and metastasis, was up-regulated by tumor tissue ischemia and hypoxia after TACE [48].

In the present meta-analysis, in-hospital mortality did not differ significantly between TACE group patients and non-TACE group patients. However, Gerunda *et al. *[25] reported that three patients died of liver failure during 2–5 months after surgery in the TACE group. Sasaki *et al. *[34] and Uchida *et al. *[29] reported that late death due to liver failure was significantly higher in the TACE group than that in the non-TACE group. Uchida *et al. *[29] considered that hepatic function impairment induced by TACE could be repaired easily in the noncirrhotic liver, but hepatic function may gradually and progressively deteriorate due to TACE in some cirrhotic patients.

This study has several limitations. First, much of the evidence comes from NRCTs that could either exaggerate or underestimate the measured magnitude of effect size [20]. To minimize this effect, we limited the analysis to well-designed studies. As a matter of fact, the estimates from overall meta-analysis were consistent with those derived from RCTs, suggesting that the magnitude of the effect was not affected by the inclusion of NRCTs. Second, significant heterogeneity was present in some outcomes. Variability in the surgeon experience and the chemoembolization schedule may have introduced potential bias. In addition, clinicopathological factors associated with recurrence, such as hepatitis status, cirrhotic liver, and tumor staging, might be another source of potential heterogeneity. The use of random-effects models partially mitigates this concern. Third, although 21 studies involving more than 3,000 patients were enrolled for analysis, funnel plot analysis revealed the sign of publication bias. This may relate to the use of published English data only. Fourth, it is important to note that 18 of the 21 studies were from Asia. This may raise a question regarding the validity of the results and applicability to other areas. Finally, although some authors have reported the efficacy of preoperative TACE for patients with advanced HCC [33] and large HCC [31,40], the results are not further estimable for subgroup analysis given the absence of data in this respect in the other studies.

## Conclusions

The updated meta-analysis represents the largest body of information currently available for assessing the role of preoperative TACE for HCC. This study demonstrates that preoperative TACE does not seem to improve the prognosis and therefore it should be prudent to recommend it as a preoperative routine procedure for resectable HCC.

## Competing interests

The authors declare that they have no competing interests.

## Authors’ contributions

YZ participated in the design and coordination of the study, carried out the critical appraisal of studies and wrote the manuscript. LW, FY, XZ, and XS developed the literature search, carried out the extraction of data, assisted in the critical appraisal of included studies and assisted in writing up. LS,YZ, and BL carried out the statistical analysis of studies. All authors read and approved the final manuscript.

## Pre-publication history

The pre-publication history for this paper can be accessed here:

http://www.biomedcentral.com/1471-230X/13/51/prepub
